# Organizational commitment, psychometric qualities and invariance of the Meyer and Allen Questionnaire for Portuguese Nurses^1^

**DOI:** 10.1590/1518-8345.2407.3021

**Published:** 2018-09-06

**Authors:** Teresa Neves, João Graveto, Victor Rodrigues, João Marôco, Pedro Parreira

**Affiliations:** 2 Doctoral student, Faculdade de Medicina, Universidade de Coimbra, Coimbra, Portugal. RN, Centro Hospitalar e Universitário de Coimbra, Coimbra, Portugal.; Centro Hospitalar e Universitário de Coimbra, Coimbra, Portugal; 3 PhD, Adjunct Professor, Escola Superior de Enfermagem de Coimbra, Coimbra, Portugal.; 4 PhD, Associate Professor, Faculdade de Medicina, Universidade de Coimbra, Coimbra, Portugal.; 5 PhD, Associate Professor, William James Centre for Research, Instituto Universitário de Ciências Psicológicas, Sociais e da Vida, Lisboa, Portugal.

**Keywords:** Nursing Staff, Personnel Loyalty, Health Human Resource Evaluation, Nursing Administration Research, Psychometrics, Validation Studies

## Abstract

**Objective:**

to evaluate the psychometric qualities of the Portuguese version of the Organizational Commitment Questionnaire for the nursing context, through confirmatory analysis and invariance, aiming to evaluate the reliability, internal consistency, construct validity and external validity of the instrument.

**Method:**

confirmatory factor analysis of the Portuguese version of the questionnaire was carried out with a sample of 850 nurses, in hospital context. The analysis was complemented using specification search. Goodness of fit was evaluated through different indices. Reliability, internal consistency and construct validity were estimated. The invariance of the model was evaluated in two subsamples of the same sample, in order to confirm the external validity of the factorial solution.

**Results:**

the refined model demonstrated good overall fit (χ^2^/d*f*=6.37; CFI=0.91; GFI=0.92; RMSEA=0.08; MECVI=0.62). The factorial structure was stable (λ:Δχ^2^(14)=18.31; p=0;193; Intercepts: Δχ^2^(14)=22.29; p=0.073; Covariance: Δχ^2^(3)=6.01; p=0.111; Residuals: Δχ^2^(15)=22.44; p=0.097).

**Conclusion:**

the simplified model of the questionnaire demonstrated adequate goodness of fit, representing a stable factorial solution. The instrument was fit to monitor and evaluate the organizational commitment of Portuguese nurses.

## Introduction

The commitment of human resources to career and organization is crucial for personal, professional and organizational development^1^.

In the organizational context, commitment is a multidimensional construct related to a psychological state associated with the affective relationship between employee and organization, the perception of a moral obligation of permanence for loyalty, and the cost/benefit associated with leaving the organization. It influences satisfaction, performance, productivity and professional investment, as well as turnover, with a strong impact on organizational behavior^2^ ^-^ ^6^.

In theoretical terms, the reference model for the analysis of organizational commitment defines the construct in three components: affective, continuance and instrumental/normative^2^ ^,^ ^7^ ^-^ ^8^. 

The Affective Organizational Commitment (AOC) is related to the emotional attachment established between employee and organization, when the employee identifies himself with the company due to compatibility with human values and common goals. Therefore, it is related to personal perception of the objective and subjective characteristics of the organization, and it is fundamental for satisfaction, professional motivation and organizational success^2^ ^,^ ^9^ ^-^ ^10^. 

Complementarily, the continuance organizational commitment (COC) is related to the employees’ perception of the cost associated with leaving the organization. Intention to quit is commonly related to the rewards system, professional recognition/appreciation, and career development opportunities. Therefore, the employees’ personal investment is adjusted to the return they expect from the organization^2^ ^-^ ^3^ ^,^ ^8^ ^-^ ^9^. 

Normative organizational commitment (NOC) is associated with the employee’s moral duty to the organization and the surplus value received, which arouse the feeling of obligation of permanence. This can promote a performance of activities with competence, according to the norms of the institution, but without great motivational involvement^2^ ^-^ ^4^ ^,^ ^8^ ^,^ ^11^. 

According to this approach, employees with strong AOC are committed and remain in the organization on their own will, without considering quitting it. Regarding the COC, which is associated with the achievement of personal objectives, the employees remain in the organization by necessity, by the lack of alternatives and by the costs associated with the change. Finally, a strong NOC results from the sense of associated duty and moral responsibility with the organization^2^ ^-^ ^3^ ^,^ ^12^. 

In theory the conceptual model is based on the independence of the three components. However, scientific research has shown that there are correlations between its dimensions. These dimensions are influenced by employees’ work experiences, and it is expected that different levels of commitment in each of the components will coexist, with a variable impact on the behavior in the work context^2^ ^-^ ^3^.

Therefore, organizational commitment arises from the joint influence of factors such as personal, structural, and performance-related characteristics and work experiences. It is also a variable that determines intention to quit, professional performance and organizational success^6^ ^,^ ^13^.

In organizational terms, and specifically in the area of health, the organizational commitment of human resources plays a key role in individual and organizational performance, in an effective and efficient manner. High levels of organizational commitment are determinant for attaining organizational objectives, and therefore can confer a competitive advantage to the health institutions. However, the socio-economic context of recent years has led to the need for cost containment, with an impact on the effective management of resources, particularly human resources. Thus, organizational and legislative changes have been necessary, which influenced the relationships of health professionals with organizations, with subsequent impact on motivation, organizational commitment, and on the quality of health care^6^ ^,^ ^11^ ^-^ ^12^ ^,^ ^14^ ^-^ ^16^.

Regarding nursing, this is particularly relevant given its privileged role in the provision of health care and considering it is the professional group most present in health systems. However, increasingly precarious employment contracts and unfavorable working conditions influence nurses’ organizational commitment and subsequent health outcomes. In Portugal, the ratio of nurses (6.58 nurses/1,000 inhabitants) is very low compared to the average of the Organization for Economic Co-operation and Development (9.16 nurses/1,000 inhabitants), with the majority concentrated in hospital care. However, the high level of training of Portuguese nurses must be highlighted, considering that most have a bachelor’s degree (4 years undergraduate course) and 22.53% have the title of specialist nurse (variable training time between 18 and 24 months)^6^ ^,^ ^12^ ^,^ ^14^ ^-^ ^15^ ^,^ ^17^. 

Scientific evidence points to the organizational commitment of nurses as predictor of performance. It is also determinant for commitment with the patient and for the development of positive behaviors, minimizing negative aspects such as conflicts and professional exhaustion and having an impact on the quality of nursing care and on patient satisfaction^6^ ^,^ ^9^ ^,^ ^12^ ^,^ ^18^ ^-^ ^19^.

The evaluation of the organizational commitment is, therefore, fundamental for its understanding. This research aims to contribute to the validation of a specific measurement instrument for the evaluation of the organizational commitment of nursing professionals.

The Organizational Commitment Questionnaire (OCQ), built in 1990 for the North American context and reviewed by the authors in 1997, includes three scales to evaluate affective, continuance and normative commitment. Its revised version presents acceptable values of internal consistency (*Cronbach’s* *alpha* (α)_AOC_=0.85; α_COC_=0.79; α_NOC_=0,73)^8^. This is one of the instruments most used internationally in scientific research to assess organizational commitment, namely in Portugal and among health professionals, especially nurses^4^ ^,^ ^13^ ^,^ ^20^ ^-^ ^22^.

In the Portuguese context, the translated version of the OCQ^3^ resulted from a translation and retroversion work, which included the development of two pre-tests, aimed at cultural adaptation. The posterior validation study, which used exploratory and confirmatory factor analysis, also demonstrated the three-dimensional structure, and the three scales had a high internal consistency (α_AOC_=0.91; α_COC_=0.91; α_NOC_=0.84), higher than original version. It should also be noted that there were problems with the adjustment of some items from the original scale to the factorial model, and therefore they were eliminated. The refined model showed goodness of fit indicators at the threshold of acceptability (chi-square to degrees of freedom (χ^2^/df)=4.17; goodness-of-fit index (GFI)=0.82; root mean square error of approximation (RMSEA)=0.08). However, regarding the confirmation of the three-component model, it has not been validated since the relationships identified between the components are not in agreement with the theoretical and empirical model, namely due to the negative correlation (*r*=-0.51) between the AOC and the CON, and the correlation (*r*=0,48) between the COC and the CON^3^. 

In this context, the present research aims to evaluate the psychometric qualities of the Portuguese version of the OCQ, for the context of nursing, through confirmatory analysis and invariance, aiming to evaluate the reliability, internal consistency, construct validity and external validity of the instrument. 

## Method

A quantitative, cross-sectional, non-experimental, validation study was carried out to evaluate the psychometric properties of the Portuguese version of the OCQ, in the context of Portuguese nursing. 

The target population includes nurses who provide direct care to patients in 71 inpatient services for general surgery, internal medicine and orthopedics of 12 public hospital units in the central and northern regions of Portugal. Data was collected between January 15^th^ and September 15^th^ 2015. 

The sample size was calculated based on a formula for the analysis of structural equations^23^ and an estimate of 255 individuals was obtained. However, given the objective of studying the psychometric properties of the OCQ, the sample selected was composed of the maximum number of participants of the target population, in order to ensure the external validity of the results and the generalization of the conclusions for the study population.

The inclusion criterion in the sample was providing direct nursing care, excluding the nurses with exclusive management functions.

The sample consisted of 850 nurses out of the 1844 questionnaires distributed (response rate of 46.10%). 

**Figure 1:**
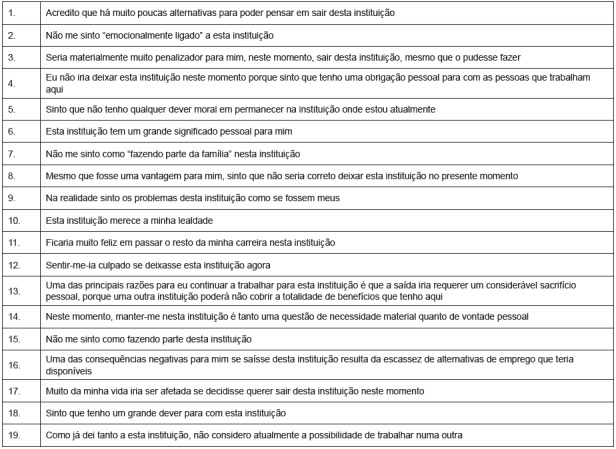
Organizational Commitment Questionnaire (OCQ)

The data collection instrument includes the Portuguese version of the OCQ and socio-demographic aspects, such as age, gender, civil status, level of education, specialized functions, length of service, employment contract, weekly hours and type of hours^3^. The OCQ is composed of 19 items, divided in three independent scales, to evaluate the organizational commitment in the affective component (6 items), continuance (7 items) and normative (6 items), according to Figure 1. Items are answered on a 7-point Likert scale, with (1) meaning “Totally Disagree” and (7) “Totally Agree”. The highest value obtained in the scales corresponds to the most evident organizational commitment dimension, considering 3.5 as the midpoint of the scale.

The descriptive analysis (measures of central tendency, dispersion and frequency) was conducted using the Statistical Package for the Social Sciences (version 22.0, SPSS An IBM Company, Chicago, IL). Items 2, 5, 7 and 15 were inverted before the factor analysis. Given the results of the previous exploratory study of the Portuguese version of the OCQ^3^, after confirming the three factors of the model, according to the original version, a confirmatory factor analysis was conducted in the AMOS software (version 22, An IBM Company, Chicago, IL) to verify the adequacy of the data to the study model. In this study, all items of the original scale were included, although some items were eliminated in the previous validation study for the Portuguese context^3^. 

Normal distribution was determined by the asymmetry (Sk) and kurtosis (Ku) coefficients, considering that |Sk| < 3 e |Ku| < 10 did not indicate significant deviations from the normal distribution, which made the analysis through the maximum likelihood method possible. The presence of outliers was evaluated by the Mahalanobis squared distance (D^2^). Omitted values were replaced by the mean of the series, given the small percentage in the study sample (less than 1.5%)^24^. 

The overall goodness of fit was assessed according to chi-square values (χ^2^), χ^2^/df, comparative fit index (CFI), goodness of fit index (GFI), root mean square error of approximation (RMSEA), P[rmsea≤0.05], 90% CI and modified expected cross-validation index (MECVI), considering acceptable values of χ^2^/df<5, CFI e GFI>0.90, RMSEA<0.08, and the lowest MECVI identifies the model with better external validity^24^ ^-^ ^26^. 

The modifications introduced for the adjustment of the model were supported by the modification indices (greater than 11; p<0.001) provided by AMOS, and by theoretical considerations^24^.

Considering the need to readjust the model in order to find the best subset of factors and reflective variables and to optimize the goodness of fit and the parsimony of the factorial solution, an additional exploratory strategy was used: a heuristic exhaustive search based on the previous analysis of the modification indices^24^.

In order to verify the external validity of the factorial model obtained, a cross-validation was performed, comparing the indices observed in the test sample with the indices obtained in another independent sample, extracted from the same population, through multi-group analysis. Therefore, the total sample was randomly divided into two approximately equal parts. The factorial invariance (configural, metric and scalar) of the measurement model was tested in both groups by comparing the free model in the two groups with a constrained model in which factor weights (loading), intercepts, residuals and variances/covariance are fixed in both groups. The statistical significance of the difference between the two models was determined by the Chi-square test^24^ ^,^ ^27^ ^-^ ^28^. 

Reliability of the construct and internal consistency were evaluated by composite reliability (CR) and α, considering values greater than 0.70 as indicators of appropriate reliability. The construct validity was determined in three subcomponents: the convergent validity, calculated by average variance extracted (AVE) for each factor, considering values greater than 0.50 as convergent validity indicators^24^ ^,^ ^26^; the discriminant validity, considered evident when the AVE value of each of two factors is equal or higher than the square of the correlation between these factors; and factorial validity, which considers the standardized factor loading (λ) and individual reliability (λ^2^), which are also indicators of the goodness of the local fit. Factor loadings greater than 0.50 and, subsequently, individual reliability greater than 0.25^24^ ^,^ ^29^ are usually considered appropriate; however, the area of social sciences sometimes considers lower values^30^. In a previous study of the OCQ, the authors propose factor loadings greater than 0.40^31^. However, in this investigation, we chose to consider factor loadings equal to or greater than 0.30 and individual reliability equal to or greater than 0.09, since previous studies, in the Portuguese context, already presented factor loadings of this order(3,32).

This study is part of a broader research on the topic of safe staffing and quality of nursing care, approved by the Board of Directors and Research Ethics Committees of the hospital institutions, as well as the Research Ethics Committee of the Faculty of Medicine of the University of Coimbra, Portugal (Proc. EC 100/2014). The participation of the nurses was voluntary, and the questionnaires were delivered personally to the head nurse of each service (who had a mediating role in the delivery and collection of the questionnaires), and then made available to all nurses. The questionnaires were completed according to availability and then delivered in a sealed envelope. Expressed informed consent was requested to the participants, ensuring compliance with ethical principles, such as anonymity and confidentiality.

## Results

The analysis of the socio-demographic characteristics reveals that the sample is predominantly female (81.86%) and aged between 22 and 59 years. Regarding educational qualifications, the most common academic degree was 4-year bachelor’s degree (89.05%), and 27.07% of the nurses had a specialization course in nursing. The most prevalent employment contract was individual (59.70%), with a working shift of 40 hours a week (86.03%) and work organized by shifts (81.71%), or roulement, as described in Table 1.

**Table 1:** Socio-demographic characteristics of the nurses studied. Central and North Regions, Portugal, 2015

Socio-demographic characteristics	M*	SD^†^	n^‡^	%^§^
Age (*years)*	36.11	±7.97		
Gender				
Male			152	18.14
Female			686	81.86
Educational Qualifications				
Bachelor’s degree (3 years degree)			10	1.19
Bachelor’s degree (4 years degree)			748	89.05
Master’s degree			80	9.52
PhD			2	0.24
Specialization				
Course			222	27.07
Job function			70	33.02
Length of service (years)	12.99	±7.80		
Less than 1 year			10	1.22
1 to 5 years			118	14.43
6 to 10 years			268	32.76
11 to 15 years			155	18.95
16 to 20 years			128	15.65
More than 20 years			139	16.99
Employment contract				
Individual employment contract			483	59.70
Public service employment contract			315	38.94
Other			11	1.36
Weekly hours				
35 hours/week			106	12.88
40 hours/week			708	86.03
Other			9	1.09
Type of hours				
Fixed (Only mornings)			131	15.98
Shifts (*roulement*)			670	81.71
Other			19	2.32

*M - Mean; †SD - Standard Deviation; ‡n - Absolute frequency; §% - Relative frequency

The descriptive analysis of the OCQ items (mean, median, mode, standard deviation, univariate asymmetry and kurtosis) shows that they present adequate psychometric sensitivity and do not present severe deviations from uni- and multivariate normality, which would hinder the factor analysis. 

**Figure 2:**
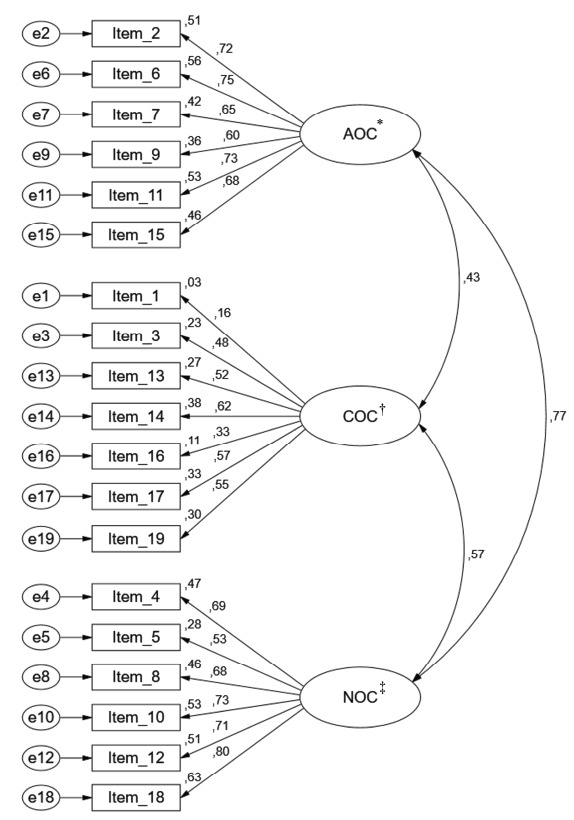
Three factor structure of the original Organizational Commitment Questionnaire (χ^2^/*df*=9.91; CFI=0.78; GFI=0.83; RMSEA=0.10; MECVI=1.84)

The confirmatory factor analysis of the three-factor OCQ model revealed a poor fit to the data obtained from the sample of 850 nurses (χ^2^/df = 9.91; CFI=0.78; GFI=0.83; RMSEA=0.10; MECVI=1.84), according to Figure 2.

The analysis of the factor loadings and individual reliabilities showed that item 1 (“*Acredito que há muito poucas alternativas para poder pensar em sair desta instituição”*), from the COC scale, presented values lower than those previously established (λ<0.30; λ^2^<0.09), suggesting its removal from the model. Regarding normality assumption, all items presented adequate values. However, there were several observations considered multivariate outliers (p_1_ and p_2_<0.001). In a conservative strategy, the analysis was conducted again after excluding eight observations, finding high D^2^ values and no evidence of improvement in the goodness of fit; therefore, these observations were kept.

*AOC - Affective organizational commitment; †COC - Continuance organizational commitment; ‡NOC - Normative organizational commitment

In order to refine the model, the analysis of the modification indices allowed to identify items that were not fit to the factor model, due to the covariance between the residuals and the latent factors and the possible existence of additional trajectories between them, specifically in items 1, 2, 9, 10, 11, 14, 15, 16, 17 and 19. In this context, given the high number of items that could present saturation in more than one factor and thus impair the clear definition of these factors, a complementary, exploratory strategy was used to evaluate the plausibility of another factorial structure, namely the heuristic specification search. According to the previous theoretical framework, the latent variables were maintained and the optional trajectories were identified, according to the previous evaluation of the modification indices. 

The exhaustive heuristic search allowed, according to the statistical criteria, to identify the most parsimonious and better fitted model (χ^2^/df=7.81; p*<*0.001). In the analysis of the disposition of the items, it was verified that the trajectory of 9, 14 and 19 (“*Na realidade sinto os problemas desta instituição como se fossem meus*”; “*Neste momento, manter-me nesta instituição é tanto uma questão de necessidade material quanto de vontade pessoal*”; “*Como já dei tanto a esta instituição, não considero atualmente a possibilidade de trabalhar numa outra*”) was altered for the NOC factor. Regarding item 1, in agreement with the result of the confirmatory analysis, the specification search revealed that it did not saturate in the COC scale, nor in any of the others. The remaining items were maintained according to the original factor solution. 

In this context, considering the theoretical framework and the disposition of the items in the original scale, we chose to remove the items 1, 9, 14 and 19. The simplified model showed a better goodness of fit (χ^2^/df=8.30; CFI=0.87; GFI=0.89; RMSEA=0.09; MECVI=0.93) compared to the original model, but was still overall poor. 

The analysis of the modification items of this simplified model showed a high correlation between the measurement errors of items 7 (“*Não me sinto como “fazendo parte da família” nesta instituição*”) and 15 (“*Não me sinto como fazendo parte desta instituição*”), from the AOC factor. Given the similarity and proximity of formulation and content between these items, item 7 was removed due to its lower factor loading. The analysis also supported the decision to remove item 10 (“*Esta instituição merece a minha lealdade*”) from the CON scale, given its correlation with other factors.

**Figure 3:**
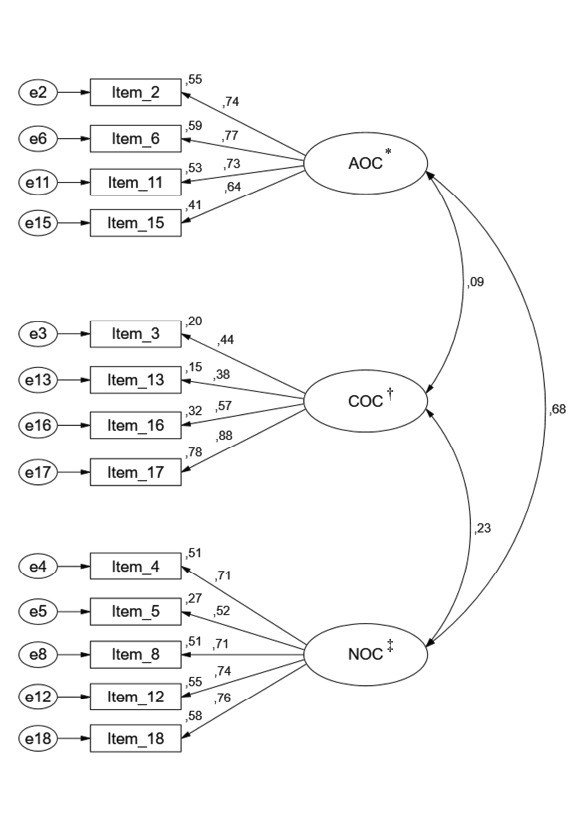
Factorial structure of the refined model of the Organizational Commitment Questionnaire (χ^2^/df=6.56; CFI =0.91; GFI=0.93; RMSEA=0.08; MECVI=0.55)

The refined model obtained an acceptable fit to the study sample in most of the indices (χ^2^/df=6.56; CFI=0.91; GFI=0.93; RMSEA=0.08; MECVI=0.55), as shown in Figure 3. As verified in this sample, the simplified model presents a better fit to the correlational structure observed between the items when compared to the original model (χ^2^_(87)_=1069.77; p<0.05); it also obtained a lower MECVI (0.55 vs. 1.84).

*AOC - Affective organizational commitment; †COC - Continuance organizational commitment; ‡NOC - Normative organizational commitment

A moderate correlation between the COA and CON scales was found (r=0.68), along with a very low correlation between the COC scale and the COA and CON scales, as shown in Figure 3. 

Reliability of the construct, evaluated by Cronbach’s alpha and composite reliability, revealed adequate internal consistency of the COA and CON scales (CC and α≥0.70), with values slightly lower for the COC scale, according to Table 2. The standardized factor loadings varied between 0.38 and 0.88 and the individual reliability of each item varied between 0.15 and 0.78 (Figure 3). 

**Table 2:** Analysis of the reliability of the construct, convergent validity and discriminant validity of the factors of the Organizational Commitment Questionnaire (refined model), for a sample of nurses from the Central and Northern Portugal regions, 2015

**Factors**	**No. of items**	CR*	**α** ^**†**^	**AVE** ^**‡**^	**ρ** ^**2§**^
**AOC** ^**||**^	**4**	**0.81**	**0.82**	**0.52**	**0.01 - 0.46**
**COC** ^**¶**^	**4**	**0.67**	**0.65**	**0.36**	**0.01 - 0.05**
NOC**	**5**	**0.82**	**0.81**	**0.49**	**0.05 - 0.46**

*CR - Composite reliability; †α - Cronbach’s alpha; ‡AVE - Average variance extracted; §ρ^2^ - Square of the correlations between factors; ||AOC - Affective organizational commitment; ¶COC - Continuance organizational commitment; **CON - Normative organizational commitment

Regarding the convergent validity, it is adequate in the AOC scale, the AVE is on the threshold of acceptability in the NOC scale, and the lowest value is in the COC scale. The comparison of the AVEs with the squares of the correlations between the factors demonstrated the discriminant validity of the scales.

The analysis of the invariance of the model in the two independent samples (test and validation) revealed adequate fit indices in the final factor solution (χ^2^/df=4.18; CFI=0.89; GFI=0.91; RMSEA=0.06; MECVI=0.82). There were no statistically significant differences in the overall fit of the model between the two samples when comparing the free model with a constrained model regarding the factorial structure, intercepts and covariance of the factors (λ: Δχ^2^(10)=16.37; p=0.090; *Intercepts*: Δχ^2^(13)=21.73; p=0.060; *Covariance*: Δχ^2^(6)=8.89; p=0.180; *Residuals*: Δχ^2^(13)=23.32; p=0.038). Thus, strong invariance is verified in both samples, confirming the external validity of the factorial structure of this reduced version of the instrument, according to Figure 3. 

## Discussion

The present study analyzed the psychometric properties of the OCQ on a sample in the context of Portuguese nursing, confirming the three-factor structure to evaluate the organizational commitment in the affective, continuance and normative components, as defined by the authors of the model^2^ ^,^ ^8^. 

Given the relevance of this instrument for the conceptualization and operationalization of the construct of organizational commitment in nursing, the present study aims to contribute with the evaluation of the validity, reliability and invariance of a model adapted to the Portuguese reality, representing a preliminary step to the development of other investigations. 

Considering the objective of assessing the psychometric qualities of the Portuguese version of the OCQ, despite the fact that a sample of 255 individuals was determined as appropriate, a sample larger than the recommended for the analysis of structural equations was used, aiming to adequately represent the population variability and conduct the analysis of invariance. 

The results of the research, supported by theoretical, semantic and conceptual frameworks, demonstrated the need to adapt the original version. This resulted in a simplified model constructed with the removal of one item from the AOC and NOC scales and three items from the COC scale. This model shows better fit to the characteristics of the sample under study when compared with the results obtained with the original model.

Previous studies have shown that it is a common practice to adapt the OCQ to simplified versions, by nested models, according to the different cultural contexts. Models with different number of items were identified^3^ ^,^ ^10^ ^,^ ^32^ ^-^ ^34^. 

In the initial work of translation and validation for the Portuguese context, two original items had already been eliminated, namely item 10, due to inadequacy to the factorial structure. This study also suggests, for future investigations, the elimination of item 19, because it is in the threshold of acceptability^3^. 

A validation study for the Brazilian context also identified item 10 as an affective component^33^. It should be pointed out that the term loyalty in this item, which is classified as normative, may be, according to some authors, an indicator of affective commitment, which is probably its interpretation in the Portuguese culture^33^ ^,^ ^35^ ^-^ ^36^. 

The divergences found between the different models may be due to the fact that the solutions resulting from the exploratory factor analysis using the orthogonal rotation of the factors consider that each item saturates only in one factor. Thus, when evaluating these models through confirmatory analysis, it is common for modification indices to identify items that are reflected in different factors, influencing the goodness of fit, a frequent occurrence in social and human sciences^24^.

The analysis of the reliability and internal consistency of the model revealed adequacy of the AOC and NOC scales, with a slightly lower value in the COC scale, but still considered adequate by some authors. These results are similar to the values of the original scale and values found in other studies^12^ ^,^ ^31^ ^-^ ^32^ ^,^ ^37^, but are lower than those of the previous study of this Portuguese version^3^. 

Regarding the construct validity, in general, the factorial loadings are higher than 0.50, with only two items on the COC scale with lower values, influencing their individual reliability and factorial validity. The convergent validity, evaluated by the AVEs, showed values on the threshold of acceptability in the COA and CON scales, and slightly lower in the COC scale, due to the high variability in the factor loadings of the items. However, discriminant validity is confirmed in all scales. 

According to some authors, factor loadings equal to or greater than 0.30 or 0.40 are acceptable in exploratory analysis in the social sciences^30^ ^,^ ^38^. However, in confirmatory factor analysis, values lower than 0.50 bring into question the factorial validity and, subsequently, the convergent validity, since they influence the value of the AVE. However, in Portugal, previous studies have already considered factor loadings of 0.35, such as in the initial validation study of the OCQ for the Portuguese context and in the validation study of the AOC and COC scales for Portuguese call center workers^3^ ^,^ ^32^. 

The low values verified in items 3 (λ=0.44) and 13 (λ=0.38) may be associated with problems of cultural interpretation resulting from the adaptation process to the Portuguese nursing context. It should be noted that item 1 was also removed from the simplified model because it has very low factor loading. 

These items belong to the COC scale, which presented the greatest need for adjustment, with the removal of three items (1, 14 and 19). Therefore, for theoretical issues, items 3 and 13 were kept in the model, since they are important to ensure the evaluation of the latent construct of continuance commitment.

All modifications associated with the COC scale may be due to the fact that, in theory, this factor can be subdivided. The two-dimensional approach consists of two factors, one related to the lack of alternatives and employment opportunities and the other to the losses that would occur by leaving the organization. However, these two factors are often strongly correlated. The scientific evidence is inconclusive on the topic of the two-dimensionality of COC, since there are studies supporting both perspectives (unidimensionality and two-dimensionality) ^39^.

In the context of the factorial structure, the authors of the scale^2^ ^,^ ^8^ postulate that the affective, continuance and normative components are independent, which is an assumption supported by several studies. However, given the evidence of strong correlation between the AOC and the NOC found in most of the studies, including this one, the pertinence of their separate use is questioned, and there actually are studies that consider only the AOC and COC scales^7^ ^,^ ^32^. 

Despite the limitations regarding construct validity, the stability of this factorial solution is emphasized, with proof of the model’s strong invariance in two independent samples. 

These results show, therefore, that the proposed model is adequate to evaluate the organizational commitment in the context of Portuguese nursing. 

However, the results obtained should be analyzed taking into account the limitations of the study, particularly in relation to construct validity and type of sampling. 

In this context, additional studies are necessary, especially with different sample units and analyzing different factorial structures, considering, in particular, the possibility of dissociation of the affective commitment scale. Further studies could identify the model most appropriate to the cultural context of Portuguese nursing.

## Conclusion

The present research contributed to the study of the psychometric qualities of the OCQ in the context of Portuguese nursing. The confirmatory factor analysis and the specification search supported the refinement of the original model. In addition, a strong invariance of the simplified three-factor model in two independent samples was found. However, some limitations on reliability and construct validity were identified, and additional studies are necessary.

Therefore, the OCQ is an adjusted instrument for monitoring and evaluating the organizational commitment of nursing human resources, in the Portuguese context.
